# Endemic Asian Chytrid Strain Infection in Threatened and Endemic Anurans of the Northern Western Ghats, India

**DOI:** 10.1371/journal.pone.0077528

**Published:** 2013-10-11

**Authors:** Neelesh Dahanukar, Keerthi Krutha, Mandar S. Paingankar, Anand D. Padhye, Nikhil Modak, Sanjay Molur

**Affiliations:** 1 Indian Institute of Science Education and Research (IISER), Pune, Maharashtra, India; 2 Zoo Outreach Organization (ZOO), Coimbatore, Tamil Nadu, India; 3 Wildlife Information Liaison Development (WILD) Society, Coimbatore, Tamil Nadu, India; 4 Department of Zoology, M.E.S. Abasaheb Garware College, Pune, Maharashtra, India; 5 Department of Biodiversity, M.E.S. Abasaheb Garware College, Pune, Maharashtra, India; 6 Conservation Breeding Specialist Group South Asia (CBSG-SA), Coimbatore, Tamil Nadu, India; Leibniz Institute for Natural Products Research and Infection Biology- Hans Knoell Institute, Germany

## Abstract

The Western Ghats of India harbors a rich diversity of amphibians with more than 77% species endemic to this region. At least 42% of the endemic species are threatened due to several anthropogenic stressors. However, information on amphibian diseases and their impacts on amphibian populations in this region are scarce. We report the occurrence of *Batrachochytridium dendrobatidis* (*Bd*), an epidermal aquatic fungal pathogen that causes chytridiomycosis in amphibians, from the Western Ghats. In the current study we detected the occurrence of a native Asian *Bd* strain from three endemic and threatened species of anurans, Bombay Night Frog *Nyctibatrachus humayuni*, Leith's Leaping Frog *Indirana leithii* and Bombay Bubble Nest Frog *Raorchestes bombayensis*, for the first time from the northern Western Ghats of India based on diagnostic nested PCR, quantitative PCR, DNA sequencing and histopathology. While, the *Bd* infected *I. leithii* and *R. bombayensis* did not show any external symptoms, *N. humayuni* showed lesions on the skin, browning of skin and sloughing. Sequencing of *Bd* 5.8S ribosomal RNA gene, and the ITS1 and ITS2 regions, revealed that the current *Bd* strain is related to a haplotype endemic to Asia. Our findings confirm the presence of *Bd* in northern Western Ghats and the affected amphibians may or may not show detectable clinical symptoms. We suggest that the significance of diseases as potential threat to amphibian populations of the Western Ghats needs to be highlighted from the conservation point of view.

## Introduction

The Western Ghats, stretching across a distance of ~1600 km along the west coast of India, is one of the 34 biodiversity hotspots [1]. This zoogeographical region is rich in amphibian diversity with 159 known species of anurans, of which 137 species are endemic to this region [2-4]. The endemism is also at higher taxonomic levels with eight genera and three families, namely, Micrixalidae, Nasikabatrachidae and Ranixalidae restricted to this region [5-7]. While new species are still being described from this area [8-10], it is suggested that out of the 137 species of endemic anurans, approximately 43% are threatened with extinction (16 Critically Endangered, 28 Endangered and 16 Vulnerable) while another 3% are Near Threatened based on their restricted distribution and threats to the populations and/or habitat [2-4]. More than 43% anurans fall under the Data Deficient or Not Evaluated categories with no information about their population status, distribution and plausible threats [2-4]. While ongoing habitat modifications caused by recreational activities, conversion to agricultural land, tourism and siltation of streams have been identified as major stressors within the Western Ghats, inadequate information is available on the diseases of the amphibians in the wild to support it as a threat to the populations [11].

Chytridiomycosis is a fungal disease caused by *Batrachochytrium dendrobatidis* (*Bd*) [12,13], which has resulted in extinctions of amphibian populations worldwide [14,15]. The fungus colonizes on the keratinous epidermal layers of amphibian skin and affects the exchange of gaseous substances and osmotic balance [16,17]. *Bd* is known to have affected more than 500 species of amphibians globally [15], and has probably resulted in the decline in the population of more than 200 species of amphibians across the globe [18,19]. Although this disease was first reported in 1997 in South America [15,20], it is suggested to have co-evolved with populations of frogs in Africa [14]. Though the actual reason for the spread and virulence of the fungus across the globe still remains in question, it is hypothesized that the influence of anthropogenic factors such as trade for research, aquarium and frog leg of species such as American Bull Frog (*Lithobates catesbeianus*) and African Clawed Frog (*Xenopus laevis*), could be one of the plausible causes for the widespread occurrence of the fungus [14,18,19,21].

*Bd* has been reported from Asia, Australia, Europe, Africa and America [19,22]. However, in Asia there are comparatively recent reports [23-30] and severe population declines have not been reported from the wild. It has been suggested that the low representation of chytrid in Asia could be because of lack of studies [31]. 

The chytrid fungus, within the Indian sub-continent, was first reported by Nair et al. [29] from Ponmudi in the southern Western Ghats, based on a single specimen of *Indirana brachytarsus* and was supported only by molecular techniques. In the present study we report the presence of chytrid fungus from three endemic and threatened anuran species, Bombay night frog *Nyctibatrachus humayuni*, Leith's leaping frog *Indirana leithii* and Bombay bubble nest frog *Raorchestes bombayensis*, from northern Western Ghats using both molecular and histo-pathological techniques. 

## Materials and Methods

### Ethics statement

Animal handling and experiments were carried out in strict compliance with Committee for the Purpose of Control and Supervision of Experiment on Animals (CPCSEA) guidelines, India. All animal experimental protocols were reviewed and approved and approval by the Institutional Animal Ethics Committee (IAEC) of Zoo Outreach Organization, India. All animals were handled in strict accordance with good animal practice as defined by Institutional Animal Ethics Committee (IAEC) of Zoo Outreach Organization, India. We also followed the guidelines for the use of live amphibians and reptiles in field research compiled by the American Society of Ichthyologists and Herpetologists (ASIH), The Herpetologists' League (HL) and the Society for the Study of Amphibians and Reptiles (SSAR). The collection of threatened species was done following the guidelines of IUCN [32]. We used non-invasive swabbing technique for sampling the fungal disease and collection of frog specimens was strictly avoided. Samples were collected from non-protected areas of Harishchandragad, Mulshi and Taleghar. In the study, only three frog specimens were collected, for which the permissions were not required, for histopathological work and to avoid any false negative results. Animals were euthanized by pithing followed by decapitation and were fixed in 4% formaldehyde. 

### Study site and sample collection

**Figure 1:**
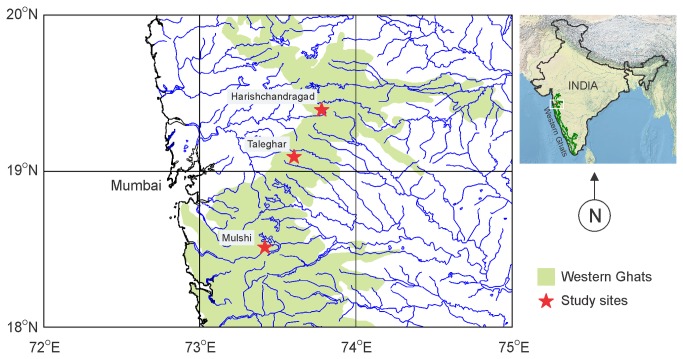
Location of the study site Harishchandragad, Maharashtra, India.

Study was conducted at three locations, namely Harishchandragad (19°23’27”N, 73°46’45”E, 1231mASL), Taleghar (19°05’20”N, 73°36’04”E, 993mASL) and Mulshi (18°30’29”N, 73°24’54”E, 663mASL), situated in the northern Western Ghats of Maharashtra, India (Fig. 1) during June 2012 – March 2013. Temperature and pH of water at the collection site was determined using Eutech® PCSTestr 35 (Fisher Scientific, USA). Swabs obtained from Medical Wire and Netting Co. (MW 113) were used. Each individual was handled using a fresh pair of gloves. A total of 32 individuals belonging to five species were sampled (Table 1). Each individual was sampled for *Bd* using the swab technique [33,34]. The swabbing for each specimen was done by applying a minimum of 60 strokes including five strokes each on both thighs, shank, toes and fingers and ten strokes on drink patch, ventrum and the region between the groin and armpit. The individuals were released back to their respective collection spots. Two adults of *Nyctibatrachus humayuni* were collected along with one adult of *Indirana leithii*, for the histological study. The specimens were preserved in absolute alcohol and presently deposited in the museum collection of Wildlife Information Liaison Development (WILD) Society, Coimbatore, India under the accession numbers WILD-13-AMP-041 to 043. Due to the potential threat of the fungus spreading through us to other locations, equipment, hands and shoes were sanitized with a mixture of 70% ethanol and Dettol® similar to the procedure described earlier [33,34]. Each specimen was handled with new gloves and the used gloves were collected in a bag for incineration at the end of the trip. 

**Table 1:** Species sampled and number of *Bd* positives detected in diagnostic nested PCR for three sites in northern Western Ghats.

Study Site	Species	Number of samples	Number of positives
Harishchandragad	*Nyctibatrachus humayuni*	8	3
	*Indirana leithii*	3	1
Taleghar	*Nyctibatrachus humayuni*	1	1
	*Raorchestes bombayensis*	3	1
Mulshi	*Nyctibatrachus humayuni*	13	2
	*Indirana leithii*	2	0
	*Euphlyctis cyanophlyctis*	1	0
	*Fejervarya caperata*	1	0

### Histology

Skin and toe clips from infected *N. humayuni* (WILD-13-AMP-041, 042) and toe clip from *I. leithii* (WILD-13-AMP-043) were used for histopathological analysis following the protocol of Berger et al. [35]. Skin and toe samples were initially stored in 4% formaldehyde and were sectioned at 5μm thickness. Sections were stained using routine hematoxylin and eosin staining method. Slides are deposited in WILD under the accession numbers WILD-13-HS-001 to 011. 

### DNA extraction, Nested PCR amplification and sequencing

DNA was extracted from swabs using the protocol specified by Goka et al. [25]. Swabs were placed in a microtube containing 200µl of lysis buffer (0.01M NaCl, 0.1M EDTA, 0.01M Tris- HCl, 0.5% Nonidet – P40, 1mg / ml Proteinase K) and the microfuge tube was vortexed for 1 min. After removing the swab, the tube was incubated at 50°C for 2 hrs and then at 95°C for 20 min. The extract was used for nested and quantitative PCR.

Diagnostic nested PCR was performed using Bd18SF1 (5’- TTT GTA CAC ACC GCC CGT CGC- 3’) and Bd28SR1 (5’- ATA TGC TTA AGT TCA GCG GG -3’) for 40 cycles followed by a second PCR using the *Bd* specific ITS1-5.8S-ITS2 gene primers Bd1a (5’- CAG TGT GCC ATA TGT CAC G-3’) and Bd2a (5’- CAT GGT TCA TAT CTG TCC AG - 3’) [25,36,37]. PCR reaction was performed in a 25µl reaction volume containing 5µl of template DNA, 2.5µl of 10X reaction buffer (100 mM Tris pH 9.0, 500 mM KCl, 15 mM MgCl_2_, 0.1% Gelatin), 3µl of 25mM MgCl_2_, 1µl of 10mM dNTP mix, 1µl of each primer, 0.5µl Taq polymerase (Promega, USA) and 11µl nuclease free water. For both sets of primer pairs the thermal profile was 10 min at 94°C, and 40 cycles of 1 minute at 94°C, 1 minute at 53°C (for BD18SF1 and BD28SR1) or 50°C (for Bd1a and Bd2a) and 2 min at 72°C followed by extension of 10 min at 72°C. PCR amplification was checked in 1% Agarose gel and positive samples were purified using the ‘Promega Wizard Gel and PCR clean up’ system (Promega, USA). The purified PCR products were sequenced using ABI prism 3730 sequencer (Applied Biosystems, USA) and Big dye terminator sequencing kit (ABI Prism, USA). Sequences were analyzed by BLAST tool [38]. These sequences have been deposited in GenBank under the accession numbers KC820805 to KC820811.

 Additional *Bd* specific ITS1-5.8S-ITS2 gene sequences of different strains from different study areas were retrieved from NCBI (http://www.ncbi.nlm.nih.gov/). Sequences were aligned using MUSCLE [39]. Neighbor joining tree was constructed using MEGA version 5 [40]. Neighbor joining tree was plotted to understand the affinity of *Bd* strain isolated in the current study with other available strains.

### Quantitative PCR

Samples which were positive for *Bd* in the nested PCR were used for quantitative PCR to analyse the extent of infection. Quantitative PCR was carried out by modifying the procedures given by Boyle et al. [41]. We used the primer pair ITS1-3 Chytr (5’-CCT TGA TAT AAT ACA GTG TGC CAT ATG TC-3’) and 5.8S Chytr (5’-AGC CAA GAG ATC CGT TGT CAA A-3’) as described by Boyle et al. [41] and performed SYBR Green I based qPCR. Conventional PCR for the validation of the specificity of the designed primers against target gene were performed in 25 μL reactions with the addition of 3 mM MgCl_2_ and employing Gotaq flexi (Promega, USA). Reactions were performed using Eppendorf master cycler (Eppendorf, Germany) under the following conditions: one cycle at 94°C for 10 min, and 50 cycles of 94°C for 15 s and 60°C for 1 min. The PCR products were analysed by running on 2% agarose gels containing ethidium bromide and visualizing for a single specific band and the absence of primer dimer products.

Real-time PCR assays were performed on Mastercycler ep realplex (Eppendorf, Germany). Assays were set up using the Kapa SYBR green qPCR kit (Kapa Biosystems, USA). Optimization of assay conditions was performed for primer, template DNA and MgCl_2_ concentrations. An optimal primer concentration of 400 nM and a final MgCl_2_ concentration of 3 mM were finally chosen for the assay under the following cycle conditions: one cycle of 95°C for 10 min for initial denaturation, and 50 cycles of 94°C for 15 s and 60°C for 1 min. Fluorescence detection was performed at the end of each extension step. Amplicon specificity was performed via dissociation curve analysis of PCR end products by increasing the temperature at a rate of 1°C every 30 s from 60 to 95°C. All samples were run in duplicates.

## Results

**Figure 2:**
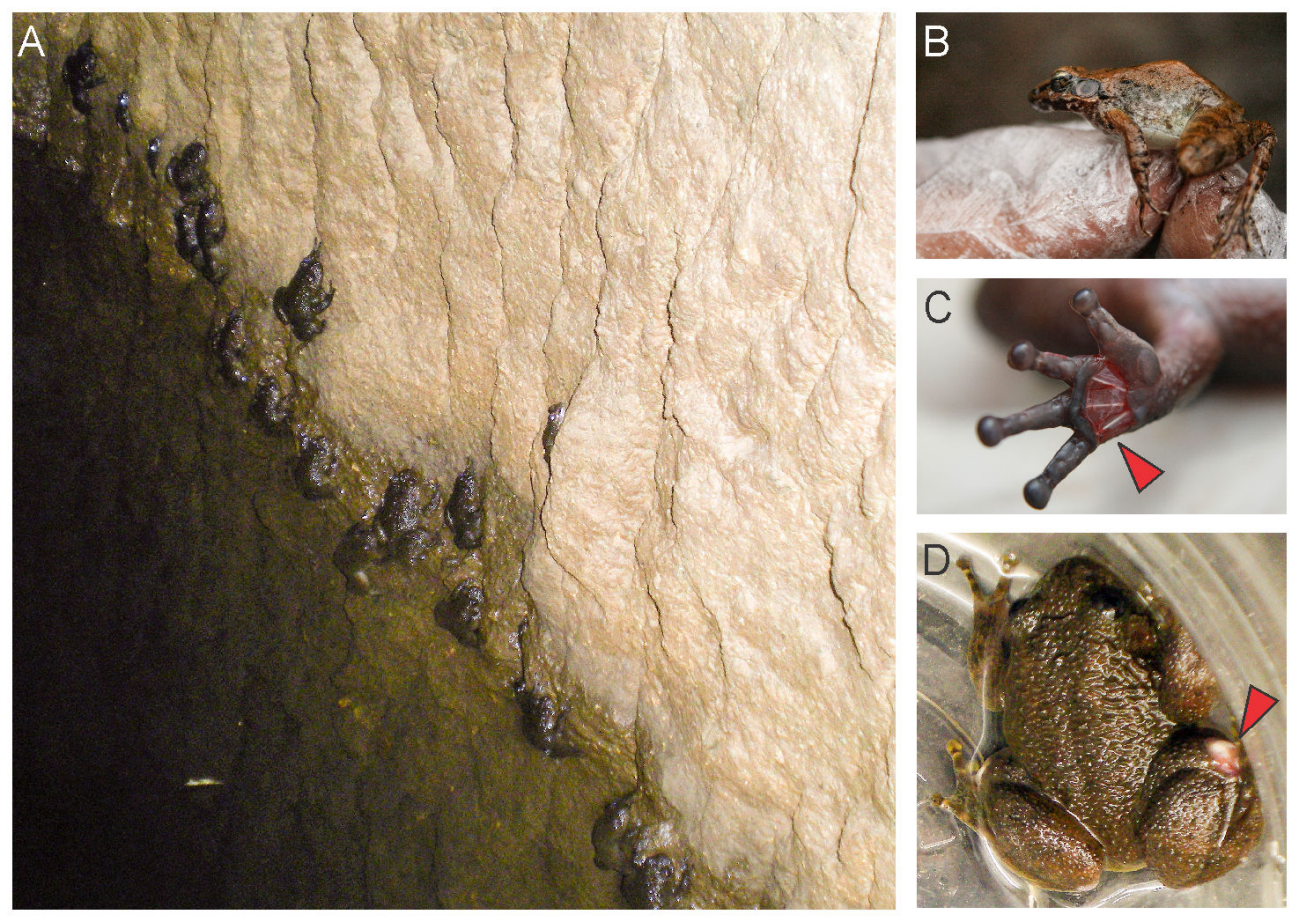
Infected specimens and habitat at Harishchandragad. (A) Habitat, (B) *Indirana leithii*, (C) *N. humayuni* skin sloughing on palm and (D) *Nyctibatrachus humayuni* skin sloughing at the joints.

Of the total 32 individuals swabbed *Bd* was detected in eight individuals using nested PCR. At Harishchandragad both *Nyctibatrachus humayuni* and *Indirana leithii* were found in a man-made water tank in the temple premises of the fort (Figure 2A). The water temperature was 21.5°C and pH was 6.9. Out of the 8 individuals of *Nyctibatrachus humayuni* that were swabbed, three tested positive for chytrid with diagnostic nested PCR, while, out of the three individuals of *Indirana leithii* swabbed only one was detected as positive for chytrid (Table 1). *Indirana leithii* individuals showed no detectable disease symptoms (Figure 2B). However, some individuals of *N. humayuni* showed skin sloughing on palm (Figure 2C) and near joints (Figure 2D). We also observed some individuals of *N. humayuni* with brownish underbelly, which is normally white in this species [8]. These individuals were sluggish and did not display immediate defense responses such as hiding under rocks or diving into the water. 

At Taleghar, *Raorchestes bombayensis* individuals that were found under rocks did not show any external disease symptoms, however, out of three individuals which were swabbed one was positive for chytrid (Table 1). Similarly, *Nyctibatrachus humayuni* individual present in the same area in a stream (pH 7.96; temperature 22.1°C), which was positive for the disease, showed no clinical symptoms. 

At Mulshi, two individuals of *Indirana leithii*, one individual each of *Fejervarya caperata* and *Euphlyctis cyanophlyctis*, which were swabbed, showed no clinical symptoms and were also negative for the disease (Table 1). Out of the thirteen individuals of *N. humayuni* that were swabbed, three were positive which also showed skin sloughing. The water temperature at this habitat was 24.5 °C and the pH was 6.8. 

**Figure 3:**
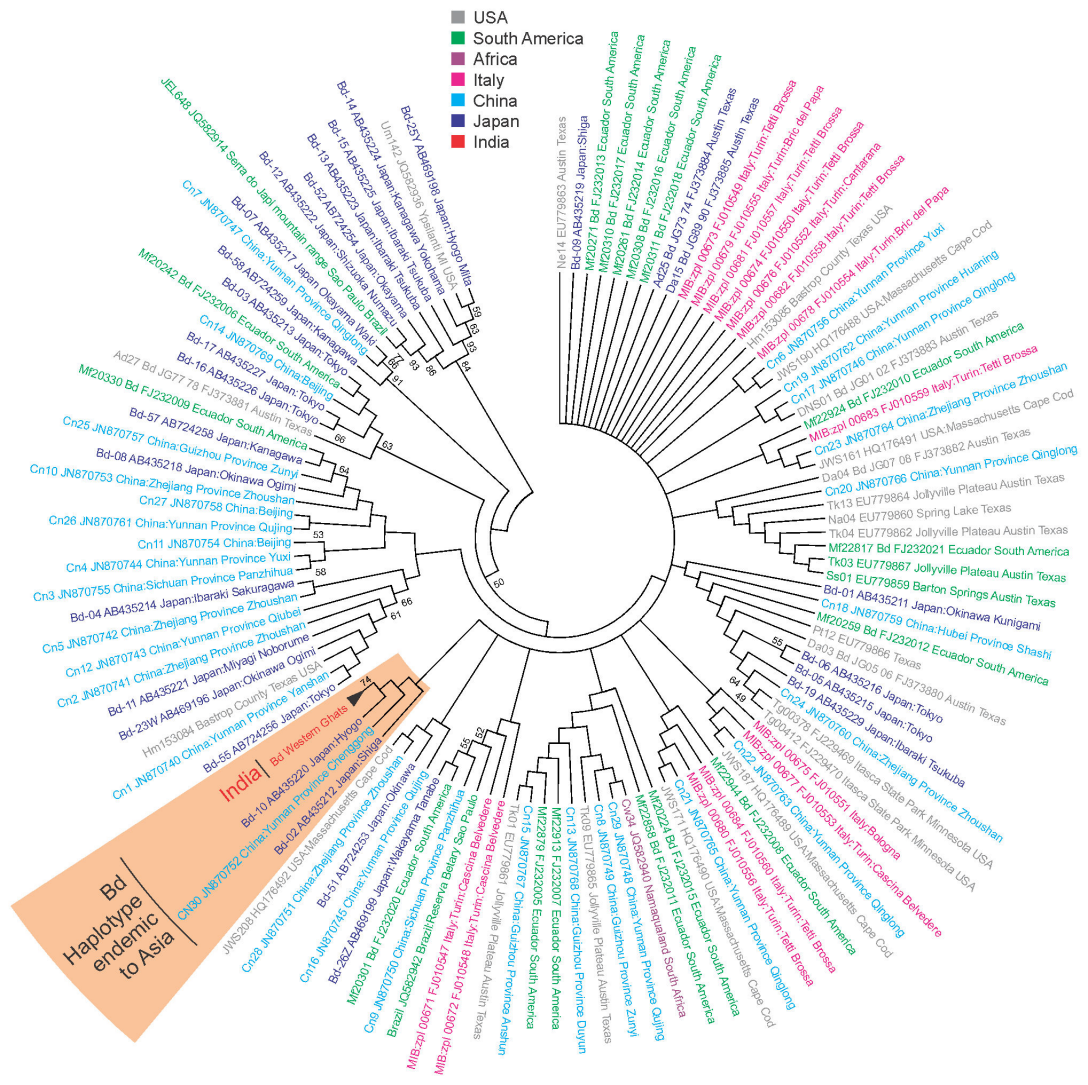
Neighbor joining tree of ITS1-5.8S-ITS2 gene sequence of *Bd*. Isolates from India showed close resemblance with the Asia endemic strains.

Sequences of *Bd* specific ITS1-5.8S-ITS2 gene from all the Western Ghats isolates were 100% identical. Sequence comparison of *Bd* isolates from the present study with all known sequences in the NCBI database revealed that the current *Bd* strain was similar to the haplotypes B and K reported from Japan and haplotype CN30 from China (Figure 3). The *Bd* haplotype from Western Ghats showed 99% similarity with haplotypes B and K of Japan and 100% similarity with haplotype CN30 from China. 

Quantitative PCRs with the SYBR assay were reproducible and gave a positive signal from 0.1 genome equivalent. The SYBR green-based assay developed in current study was as sensitive as the TaqMan assay described by Boyle et al. [41]. The primers did not form the primer dimer (assessed by gel and melting curve analysis). The fluorescence threshold for experiments varied slightly, but was chosen at a point early in each amplification reaction where the standard curve gave the highest R^2^ value (always > 0.99). The detection limit with the SYBR green assay was 0.1 zoospore genome equivalents. In this study genome equivalents below one were considered as negative. We detected low Genomic Equivalents (GE) in all of the eight chytrid-positive samples. The GE ranged from 1.80 to 13.00 with the mean GE of 5.36 and standard deviation of 3.15.

**Figure 4:**
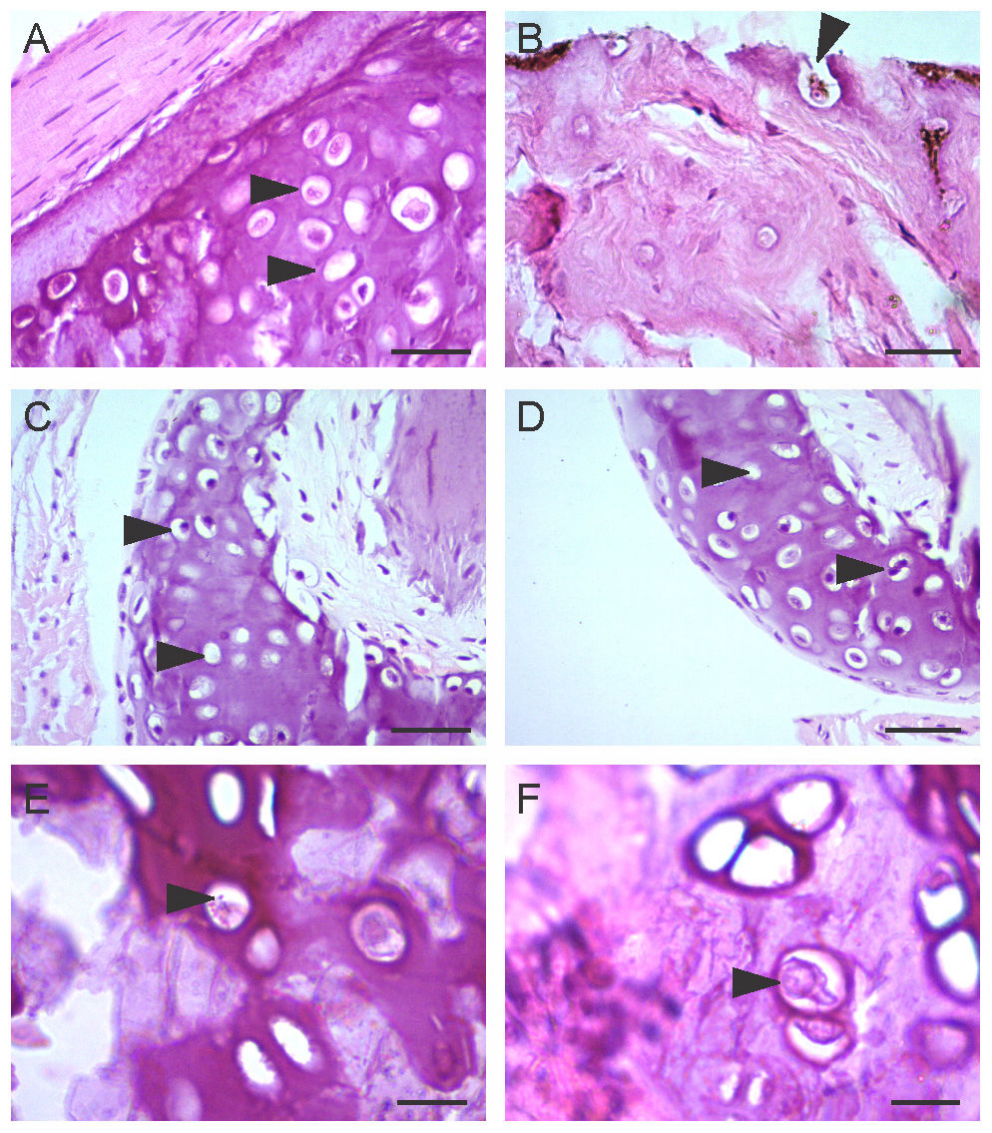
Histological sections of skin and toe clip showing the lesions caused by *Bd*. (A, B) belly skin of *Nyctibatrachus humayuni* showing filled and empty zoosporangia (C) toe disc of *Nyctibatrachus humayuni*, (D) toe disc of *Indirana leithii* and (E,F) details of skin of Nyctibatrachus humayuni. Scale bar is 50μm in A-D and 25μm in E-F.

Histopathological study of *N. humayuni* skin (Figure 4A, B) and *N. humayuni* toe (Figure 4C) and *I. leithii* toe (Figure 4D) collected from Harichandragad showed typical lesions of *Bd* infestation, where in the epidermal and sub-epidermal layers showed presence of numerous vacuole-like empty zoosporangia, pot-shaped or spherical zoosporangia with a discharge tube and zoospores (Figure 4A-D). At higher magnifications zoosporangia containing zoospores could be seen (Figure 4E). We also observed the discharge tube like structure at higher magnification (Figure 4F). The zoosporangia were found to be pre-dominantly embedded within the layers of the stratum corneum or stratum granulosum. 

## Discussions

The first report of chytrid fungus from Ponmudi in the southern Western Ghats [29] is around 1000 km south of the current reports from the northern Western Ghats. The previous report [29] was a single positive result from *Indirana brachytarsus* toe clip and was based on diagnostic and quantitative PCR. On the contrary, the present report from *Nyctibatrachus humayuni, Indirana leithii* and *Raorchestes bombayensis* is based on eight positive reports confirmed by employing diagnostic nested PCR, quantitative PCR, DNA sequencing and histopathology, which confirms the presence of *Bd* in the Western Ghats.

*Nyctibatrachus humayuni* individuals from all three sites were positive for *Bd* and showed clinical symptoms such as skin sloughing, browning of ventral side and sluggishness. However, *Indirana leithii* and *Raorchestes bombayensis* did not show any detectable clinical symptoms. Thus, our findings also support other reports [25,42] that the *Bd* infected species may or may not display clinical symptoms. A common character for all the three species of frogs infected with chytrid is the presence of dilated discs on finger and toe tips. We suspect that the prevalence of the disease in these organisms could be due to the higher surface area provided by the discs and keratinisation based on earlier reports [43]. At Harishchandragadh, although both *N. humayuni* and *I. leithii* were present in the same man-made water tank, the clinical symptoms were seen only in *N. humayuni*. Even though, the exact reason for this difference in disease symptoms is difficult to pin point, some ecological and anatomical observations could be attributed to them. *Indirana leithii* has more mucous secretion than *N. humayuni* and it is already known that mucous forms the first line of defense [44]. Further, the skin of *N. humayuni* is thicker and more wrinkled as opposed to *I. leithii*, which could help in chytrid growth and development of microsporangium tube [44]. Another reason could be due to the predominantly aquatic and group living nature of *N. humayuni*, which might be aiding the spread of the disease more effectively. The skin sloughing in *N. humayuni* could therefore be attributed to an immune response to heavy infection as suggested by Davidson et al. [45]. Despite the presence of clinical symptoms the quantitative PCR only detected low copies of fungal DNA. One reason for this could be attributed to shedding of skin and the release of microsporangium (as evident from the empty vacuoles in the histopathological work).

 The water temperature at the habitat from where the infected amphibians were collected ranged from 21.5 °C to 24.5 °C and the altitude ranged from 663mASL to 1231mASL. In Asian countries the range of temperatures for Bd infections are not available, however, Skerratt et al. [18] have suggested that *Bd* is pathogenic and virulent at temperature between 12 to 27 °C. Further, Swei et al. [22] have shown that in Asia the altitude at which *Bd* infection occur ranges from 330 mASL in the Philippines to 1949 mASL in Indonesia. Our observations of temperature and altitude at which *Bd* was detected, therefore, lie in the previous known limits. 

 Sequencing of *Bd* specific ITS1-5.8S-ITS2 ribosomal gene of seven isolates from the current study showed high similarity with other *Bd* haplotypes that have been considered as endemic to Asia. The *Bd* haplotype from Western Ghats showed 99% similarity with haplotypes B and K of Japan, which is known to infect the Japanese endemic salamander *Andrias japonicus* [25], and 100% similarity with haplotype CN30 from China, which has been found to infect an endemic frog *Babina pleuraden* [30]. Goka et al. [25] found haplotypes B and K to have infected the endemic salamander and these haplotypes were genetically different from all other known *Bd* haplotypes isolated from other amphibians of Japan and exotic amphibians in trade. Bai et al. [30] found another haplotype CN30 on an endemic anuran, which was genetically similar to haplotypes B of Japan. Bai et al. [30] suggested that it is highly unlikely that the Japanese and Chinese haplotypes have been exchanged because of pet and/or food trade and it is therefore likely that these three haplotypes are basal clades of *Bd* endemic to Asia. Our finding of *Bd* haplotypes similar to the Japenese B and K and Chinese CN30 haplotypes furthers the distribution of these haplotypes within the Indian subcontinent as well. Further, in accordance to Bai et al. [30] we believe that it is unlikely that this *Bd* haplotype has been introduced due to pet trade. A distinct clade of this Asian *Bd* haplotype (Figure 3) suggests the presence of an Asian lineage of *Bd*, which indicates that the current report of *Bd* haplotype could be a native infectious fungal strain.

 Although *Bd* haplotype in the current study is Asian endemic and we suspect it to be a native infectious fungal strain, the finding is still alarming for the native amphibian fauna and its conservation. Firstly, we do not know the virulence of the current strain and secondly, it is possible that the fungal infection may manifest in the near future through increased stressors such as organic and inorganic pollution, which might increase the virulence of the fungal strain and/or decrease the immunity of amphibian host. Both these concerns warrant further research on amphibian diseases in the Western Ghats and their effects on endemic and threatened amphibian species. It is essential to note that lack of previous reports on the population level effects of amphibian diseases does not necessarily suggest absence of such incidences. Rather, it could be attributed to lack of systematic studies and scientific documentation [31].

 Currently all three species *Nyctibatrachus humayuni, Raorchestes bombayensis* and *Indirana leithii* are assessed as Vulnerable in the IUCN Red List of Threatened Species based on restricted distribution and severely fragmented habitat with several threats to the habitat including tourism, habitat alterations, water pollution and siltation of streams [46-48]. However, there has not been any report of disease in any of these species. Our findings of chytrid fungus infection leading to skin sloughing in *N. humayuni* raises a concern that this fungal infection can pose a plausible threat to the populations of this endemic species in the Western Ghats in the future. Even though *Indirana leithii* and *Raorchestes bombayensis* did not show any pathogenic symptoms, further studies are required for assessing the effect of this fungal disease on the populations of these threatened and highly restricted species as well. Further studies on *Bd* ecology are needed to clarify the mechanism of *Bd* spread to new territories and amphibian species. Thus, in the case of Western Ghats amphibians there is an urgent need to reevaluate the importance of the disease in conservation assessment of the species and for developing conservation actions.
